# Novel biomarker identification for oral squamous cell carcinoma development in nonsmoker, nondrinker, and nonchewer patients using third-generation sequencing of oral microbiome

**DOI:** 10.1080/20002297.2025.2565452

**Published:** 2025-10-02

**Authors:** Wei-Ni Lyu, Cheng-Ying Shen, Yung-Hua Lee, Shin-Kuang Chen, Eric Y. Chuang, Pei-Jen Lou, Mong-Hsun Tsai

**Affiliations:** a Institute of Biotechnology, National Taiwan University, Taipei, Taiwan; b Center for Biotechnology, National Taiwan University, Taipei, Taiwan; c Graduate Institute of Biomedical Electronics and Bioinformatics, National Taiwan University, Taipei, Taiwan; d Department of Otolaryngology, National Taiwan University Hospital, Taipei, Taiwan

**Keywords:** Oral squamous cell carcinoma, novel microbial biomarkers, nonsmoker nondrinker nonchewer, third-generation sequencing, full-length 16S rRNA, host–microbe interaction

## Abstract

**Background/Objective:**

Oral squamous cell carcinoma (OSCC) in patients without tobacco, alcohol, or betel-quid habits is poorly understood and difficult to detect early. This study aimed to identify microbial biomarkers specific to this habit-free population using third-generation sequencing (TGS).

**Patients/Materials and methods:**

Twenty-seven habit-free OSCC patients were recruited at National Taiwan University Hospital (NTUH). Paired tumor and adjacent normal tissues were collected with informed consent and NTUH Research Ethics Committee approval (IRB 201902080RINC, 201304078RIND). Full-length 16S rRNA sequencing (PacBio Sequel IIe) was processed with DADA2 and SILVA. Biomarkers were identified using sparse partial least squares discriminant analysis (sPLS-DA) and random forest with cross-validation, and validated against three public OSCC cohorts.

**Results:**

A three-species panel—*Eikenella corrodens*, *Slackia exigua*, and *Eggerthia catenaformis*—discriminated tumor from normal tissues (AUC = 0.905 training; 0.733 testing). Functional and network analyses showed tumor-enriched taxa forming pro-inflammatory clusters linked to lipid and glutamine metabolism, while commensals correlated with homeostatic pathways. Cross-cohort comparison confirmed this panel's specificity to habit-free OSCC.

**Conclusions:**

Using TGS, we revealed distinct microbial signatures in habit-free OSCC that may aid early diagnosis and underscore the role of microbiome–host interactions in carcinogenesis.

## Key Messages

• Third-generation sequencing enabled species-level identification of microbial biomarkers in habit-free OSCC.
• A three-species panel (*E. corrodens*, *S. exigua*, *E. catenaformis*) discriminated tumor from normal tissues with high accuracy.
• Tumor-associated microbes were linked to pro-inflammatory metabolic pathways, suggesting microbiome-driven carcinogenesis.

## Introduction

Oral squamous cell carcinoma (OSCC) accounts for approximately 90% of all oral cancer [1]. Tobacco smoking and alcohol consumption are the predominant aetiological factors in Western populations [2–4], whereas betel-quid chewing predominates in South and Southeast Asia–including Taiwan [3], where cultural practices drive one of the world's highest male OSCC incidence rates [5]. Betel quid typically consists of areca nut wrapped in betel leaf, often combined with slaked lime and tobacco, and is widely chewed as a stimulant.

Despite the well-established links between OSCC and carcinogens such as tobacco, alcohol, and betel quid, a subset of patients with no history of these habits–referred to as three-habit-free (THF) patients–still develop OSCC [4–6]. This phenomenon suggests the involvement of additional, nontraditional aetiological factors. Increasing evidence implicates the oral microbiome as a key player in tumour development through mechanisms such as chronic inflammation, mucosal barrier disruption, biofilm formation, and the production of genotoxic metabolites [7–13].

The advent of third-generation sequencing (TGS) has enabled species-level resolution of microbial communities through full-length 16S rRNA gene sequencing, offering a significant advantage over next-generation sequencing (NGS), which typically targets partial regions and resolves taxonomy only to the genus level [14–18]. This enhanced precision allows for a deeper understanding of how specific bacterial species modulate host pathways–including lipid metabolism, cytokine signalling, and redox homoeostasis [14,16,18],–that are critical to oral tumour pathogenesis. These host–microbe interactions may shape the tumour microenvironment and promote epithelial transformation in individuals without conventional risk exposures.

Building on these advantages, we applied full-length 16S rRNA sequencing to characterise the oral microbiome in 27 THF OSCC patients by comparing matched tumour and adjacent normal tissues. Given the lack of publicly available TGS datasets for habit-free OSCC, we benchmarked our candidate species-level microbial biomarkers against publicly available NGS-derived OSCC cohorts characterised by traditional risk factors to confirm their specificity. Our objectives were to (1) identify microbial biomarkers capable of discriminating malignant from healthy mucosa at the species level; (2) infer their roles in inflammatory and metabolic pathways via co-occurrence network construction and KEGG functional analyses; and (3) validate their exclusivity through cross-cohort comparison. By integrating precise taxonomic profiling with pathway analyses and cross-cohort validation, we uncover novel microbial contributors to habit-free OSCC and pave the way for microbiome-targeted diagnostics and prevention strategies.

## Materials and methods

### Study design and sample collection

Twenty-seven OSCC patients without any history of tobacco use, alcohol consumption, or betel-quid chewing were recruited at National Taiwan University Hospital (NTUH) between February 2017 and August 2020. The cohort was 63.0% male, with a mean age of 53.5 ± 13.9 years; 55.5% presented with late‐stage disease (stage III–IV), and 88.9% had primary tumours on the tongue (Table 1). All patients were followed for three years. The study was approved by the NTUH Research Ethics Committee (IRB 201902080RINC and 201304078RIND), and all participants provided written informed consent. Exclusion criteria included multiple cancer types and antibiotic use within three months. Prior to surgery, patients rinsed with sterile normal saline, and paired samples were collected from tumour lesions (*n* = 27) and adjacent clinically normal mucosa (*n* = 27). Diagnosis was confirmed by magnetic resonance imaging and histopathology [19], and staging followed AJCC seventh edition guidelines [20]. All specimens were stored at –80 °C until processing.

**Table 1.** Clinical characteristics of 27 patients with oral squamous cell carcinoma (OSCC).

Variable	All patients (%)
Number of patients	27
Age (years)	
Mean ± SD	53.5 ± 13.9
Gender	
Male	17 (63.0%)
Female	10 (37.0%)
Stage	
I	8 (29.6%)
II	4 (14.9%)
III	7 (25.9%)
IV	8 (29.6%)
Alcohol drinking	None
Betel quid chewing	None
Cigarette smoking	None
Site	
Floor of mouth	1 (3.7%)
Retromolar area	1 (3.7%)
Tongue	24 (88.9%)
Gum	1 (3.7%)

### DNA extraction, library construction, and third-generation sequencing

Bacterial DNA was extracted using the QIAamp DNA Mini Kit following Lyu et al. [21]. Full-length 16S rRNA genes (V1–V9 regions) were amplified with primers 27F and 1492 R using KAPA HiFi HotStart ReadyMix (Kapa Biosystems) over 27 cycles (95 °C 30 s, 57 °C 30 s, 72 °C 30 s), with a final extension at 72 °C for 5 min. Amplicons were purified (GenepHlow™ Gel/PCR Kit), quantified by Qubit, pooled (600 ng total), and converted to SMRTbell libraries (PacBio Express Template Prep Kit 2.0). Sequencing was performed on a PacBio Sequel IIe system using SMRT 8 M cells and chemistry v2. Initial processing used SMRT Link v11.0.0

### Bioinformatic analysis

Raw PacBio Sequel IIe reads were processed with DADA2 v1.26.0 for adapter and primer trimming, chimera removal, and representative sequence selection [22]. A total of 722,650 high-quality reads (mean 13,382 reads/sample; base call accuracy >99.999%) were retained. Representative sequences were clustered into Amplicon Sequence Variants (ASVs) at 100% similarity and taxonomically classified using a pre-trained Naive Bayes classifier based on the SILVA, GTDB, and RefSeq + RDP databases [16,17,23,24] following PacBio's recommended pb-16S-nf pipeline (https://github.com/PacificBiosciences/pb-16S-nf). Downstream visualisation was performed as described by Lyu et al. [21].

### Microbial feature selection and network analysis

Sparse partial least squares discriminant analysis (sPLS-DA) was conducted in mixOmics v6.20.0 to identify ASVs that best discriminated tumour from matched normal samples. Discriminative ASVs identified by sPLS-DA were subsequently used for functional prediction and downstream network analysis.

To explore microbe–function associations, Spearman correlation coefficients (|r| > 0.3, *p *< 0.05) were calculated between sPLS-DA–selected taxa and predicted KEGG pathways using R 4.2.3's stats package [25], and networks were visualised with corrplot v0.92 and Cytoscape v3.9.1 [26].

### Predictive modelling of OSCC

For diagnostic evaluation, the full dataset (*n* = 54 samples) was randomly partitioned into 80% training and 20% testing sets. A random forest (RF) model was trained in caret v6.0-90 package, with repeated 10-fold cross-validation (five repeats) applied within the training set to optimise model performance and reduce overfitting [27]. Model performance was assessed by receiver operating characteristic (ROC) analysis, including area under curve (AUC) and accuracy, using pROC v1.18.0, with 95% confidence intervals. AUC quantifies the model's ability to distinguish between classes, with a value of 1.0 indicating perfect classification and 0.5 representing no discriminative power. Pearson's chi-squared test was applied to assess associations between key taxa and clinicopathological variables.

### Public OSCC dataset processing

Publicly available 16S rRNA sequencing datasets of OSCC were retrieved from the National Centre for Biotechnology Information (NCBI) Sequence Read Archive (SRA) for cross-cohort validation. These included OSCC cohorts from individuals with traditional smoking, alcohol, and betel-quid use (hereafter abbreviated as WTH, for ‘with the three habits’): PRJNA813634 (V4 region; *n* = 32), PRJNA597251 (V3-V4 region; *n* = 48), and PRJNA362794 (V4-V5 region; *n* = 80) [25,28,29], each comprising paired tumour and adjacent normal samples. The V3, V4, and V5 regions are hypervariable segments of the 16S rRNA gene commonly used for genus-level profiling [30,31]. Because no public TGS datasets from THF OSCC patients are currently available, direct full-length TGS-based comparison across cohorts was not feasible. To address this, the V4 region was extracted in silico from our full-length 16S rRNA PacBio reads to match the region targeted in the public datasets. The V4 region was selected because it is the only hypervariable region shared across all three WTH cohorts and is supported by a pre-trained Naive Bayes classifier in QIIME2 [32], enabling consistent and standardised taxonomic resolution. All datasets–including the in silico V4-trimmed THF data and the raw NGS reads from public cohorts–were reprocessed using QIIME2 v2021.2, including primer trimming, quality filtering, and ASV inference via DADA2. Taxonomic classification was performed using the SILVA 138 database (99%) to minimise batch effects and standardise annotation. After filtering, 71 normal and 78 tumour samples were retained across the WTH cohorts, resulting in 629 high-confidence ASVs. Differential abundance analysis (|log₂ fold change|>1), followed by a Venn diagram–based comparison, was used to identify taxa uniquely enriched in the THF OSCC group relative to the WTH cohorts.

## Results

### Microbial diversity and community structure

**Figure 1.**
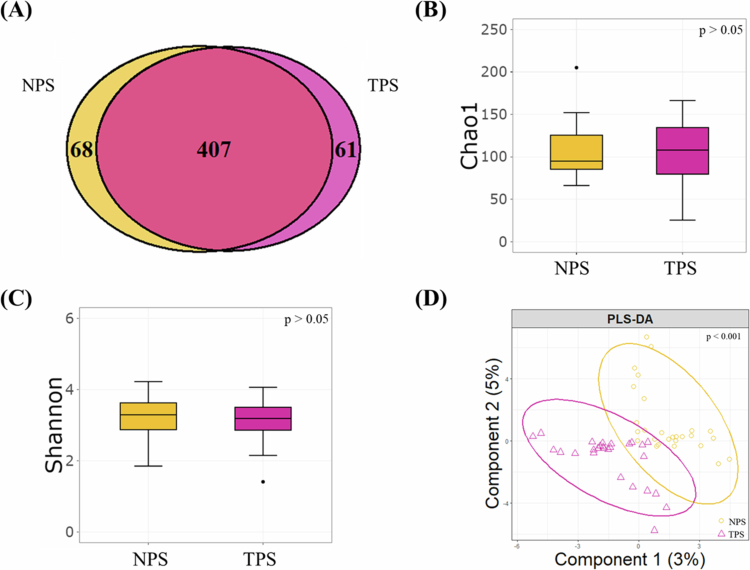
Microbial diversity of oral microbiomes between NPS and TPS samples. (A) Venn diagram showing the overlap of amplicon sequence variants between NPS and TPS. (B) Alpha diversity assessed by the Chao1 index in NPS versus TPS samples. (C) Alpha diversity assessed by the Shannon index in NPS versus TPS samples. (D) sPLS-DA score plot illustrating the distinct microbial community structures between NPS and TPS.

After DADA2 filtering, 536 high‐confidence ASVs were recovered across all samples, with 407 shared between normal paired samples (NPS) and tumour paired samples (TPS) groups and 61 ASVs unique to TPS (Figure 1A). Rarefaction curves reached plateaus for all samples, indicating sufficient sequencing depth (Figure S1). Alpha diversity metrics (Chao1 and Shannon indices) did not differ significantly between NPS and TPS (all *p *> 0.05; Figure 1B–C), while sPLS‐DA demonstrated clear separation of the two groups (*p *< 0.001; Figure 1D).

Analysis of the relative abundances revealed that, excluding low‐abundance ASVs, the top three bacterial families in both NPS and TPS samples were *Neisseriaceae*, *Streptococcaceae*, and *Pasteurellaceae*; at the genus level, *Neisseria*, *Streptococcus*, and *Peptostreptococcus* dominated (Figure S2A–B).

### Species-level differential abundance

**Figure 2.**
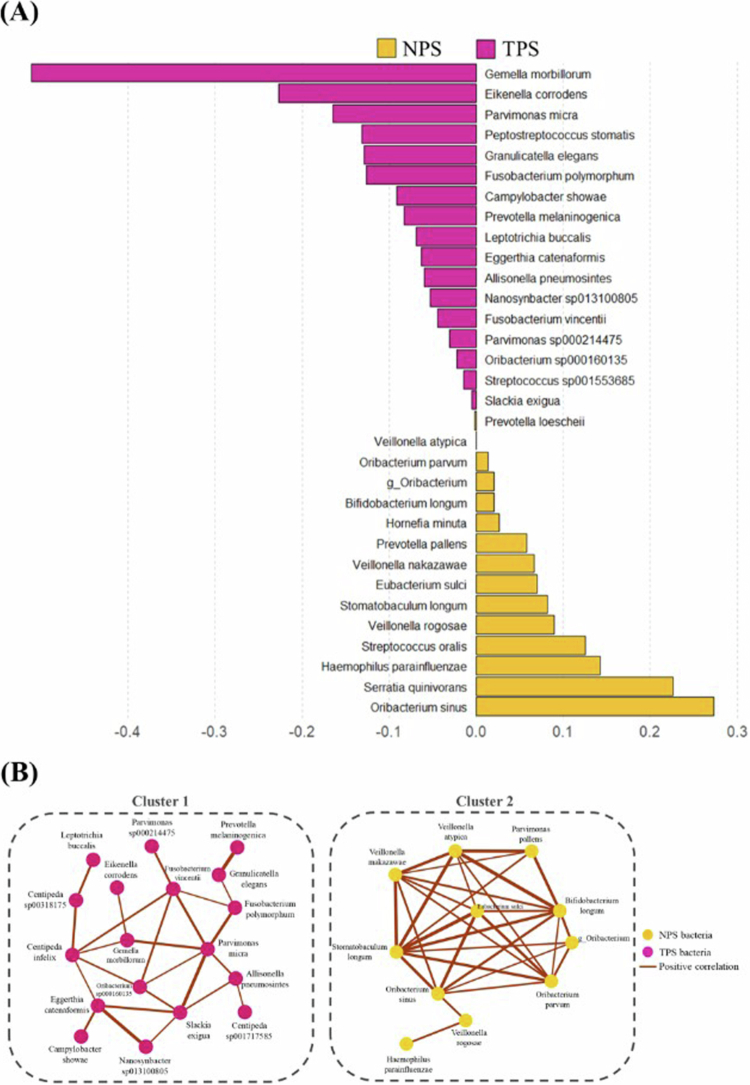
Differential abundance analysis (DAA) and correlation network analysis of the microbiome in NPS and TPS samples. (A) Differential abundance of microbiome composition between NPS and TPS samples. In the NPS group, the contribution of each bacterium to this dimension decreases from the bottom to the top, while in the TPS group, it decreases from the top to the bottom. In DAA, contribution plots generated from sPLS-DA highlight the features of amplicon sequence variants represented in the first component. (B) Correlation network analysis of the microbiome between NPS and TPS samples. In correlation network analysis, the thickness of the lines in the network represents the strength of the correlation between the microbial species. Brown lines indicate positive correlations. The threshold of positive correlation is defined as *r* > 0.3 and *p *< 0.05.

sPLS‐DA identified 32 discriminative ASVs that effectively differentiated TPS from NPS samples (Figure 2A). Among the 17 tumour‐enriched species identified by sPLS-DA (Figure 2A), three species－*Eikenella corrodens*, *Slackia exigua*, and *Eggerthia catenaformis*－ were prioritised based on an objective two-step process. First, we applied a RF classifier to all discriminative ASVs and determined the optimal feature set by maximising classification performance in the training dataset. This data-driven approach yielded the three species as the minimal panel with the highest accuracy and AUC. Second, in an independent cross-cohort comparison, these species were found to be uniquely enriched in THF OSCC and absent in WTH OSCC cohorts, as determined through sequential feature selection and validation. Although some selected species, such as *S. exigua*, exhibited relatively low abundance, their inclusion was driven by strong classification performance rather than frequency alone. This highlights the utility of multivariate selection approaches in identifying informative biomarkers that may not be the most abundant but are biologically or clinically relevant. Conversely, several commensal taxa were enriched in the NPS, including *Prevotella pallen*, *Streptococcus oralis*, and *Oribacterium sinus* (Figure 2A).

We conducted a Spearman correlation network analysis to explore microbial interactions, employing a threshold of |*r*| > 0.3 and *p *< 0.05 for robustness. The correlation network revealed two distinct co-occurrence clusters (Figure 2B). Cluster 1 consisted primarily of tumour-associated taxa, including *E. corrodens*, *S. exigua*, *Parvimonas micra*, and *Gemella morbillorum*, which exhibited strong positive interconnections, suggesting a potential tumour-promoting microbial community. In contrast, Cluster 2 comprised taxa enriched in NPS samples, displaying positive correlations among commensal species, likely reflecting a stable microbial network associated with mucosal health.

### Host–microbe functional network analysis

**Figure 3.**
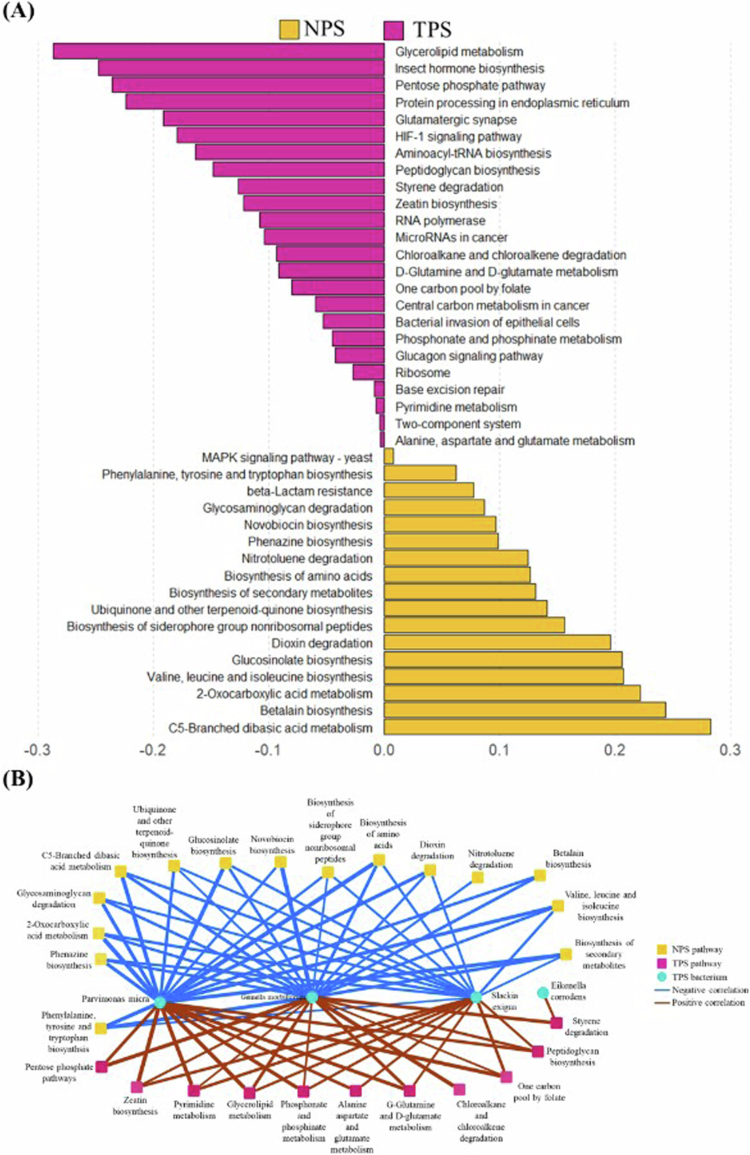
Predicted functional differences and microbial associations in TPS and NPS samples. (A) KEGG pathways enriched in tumour (TPS, magenta) and normal (NPS, yellow) tissues, identified by sparse PLS-DA. Tumour-associated pathways include lipid metabolism, protein processing, and cancer-related signalling. (B) Co-occurrence network showing correlations between dominant microbes (circles) and KEGG pathways (rectangles). Positive and negative associations are shown in brown and blue, respectively; edge thickness corresponds to correlation strength.

To further investigate the functional implications of the tumour microbiome, we performed pathway-based analyses and visualised their associations in Figure 3. This figure illustrates both the predicted functional differences between tumour and normal sites and their associations with key microbial taxa.

Spearman correlation networks (|*r*| > 0.3, *p *< 0.05) between the 32 key ASVs and predicted KEGG pathways revealed two distinct modules: a tumour-associated module of 18 ASVs linked to 24 pathways enriched in TPS samples, and a normal‐associated module of 14 ASVs associated with 17 pathways enriched in NPS samples (Figure 3A).

The bacteria–pathway correlation network revealed strong positive associations between tumour-associated taxa–*E. corrodens* and *S. exigua*, *P. micra*, and *G. morbillorum–*and TPS-enriched pathways, including glycerolipid metabolism, peptidoglycan biosynthesis, and D-glutamine and D-glutamate metabolism (Figure 3B). Conversely, these species were negatively correlated with NPS-enriched KEGG pathways such as glycosaminoglycan degradation and amino acid biosynthesis. These findings suggest that tumour-associated microbes may contribute to metabolic reprogramming linked to tumour progression, while commensal bacteria in normal mucosa are associated with pathways that support mucosal homoeostasis.

### OSCC prediction

**Figure 4.**
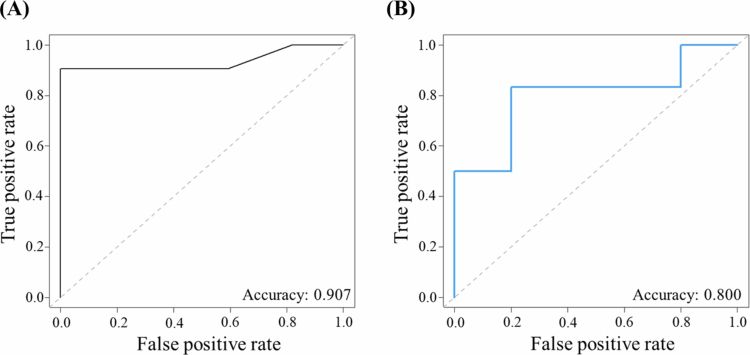
Performance evaluation of OSCC prediction. (A) ROC curve representing the performance of the RF model using three microbial features on the training dataset (accuracy = 0.907, AUC = 0.905 (95% CI: 0.819−0.991)). (B) ROC curve depicting the performance of the RF model using three microbial features on the testing dataset (accuracy = 0.800, AUC = 0.733 (95% CI: 0.449−1.000)).

To evaluate the diagnostic performance of sPLS-DA–nominated taxa (Figure 2A), we trained RF classifiers on the full dataset (*n* = 54) partitioned into 80% training and 20% hold-out testing. Within the training set, RF models were fit with repeated 10-fold cross-validation (five repeats) to optimise hyperparameters and select the smallest species subset with maximal AUC. A three-species panel–*E. corrodens*, *S. exigua*, and *E. catenaformis–*achieved the best discrimination between TPS and NPS samples. Cross-validated training performance was AUC 0.905 (95% CI: 0.819–0.991) with accuracy 0.907 (Figure 4A). On the independent test set, the model achieved AUC 0.733 (95% CI: 0.449–1.000) and accuracy 0.800 (Figure 4B).

We further explored associations between the abundance of these microbial biomarkers and clinicopathological parameters. Pearson's chi-squared tests revealed that *E. corrodens* abundance was significantly higher in cancer tissues (*p *< 0.05), supporting its association with cancer status. Moreover, *S. exigua* (*p *< 0.01) and *Eggerthia catenaformis* (*p *< 0.05) were significantly more abundant in male patients (Table S1).

### Comparison of microbial profiles between THF and WTH OSCC cohorts

**Figure 5.**
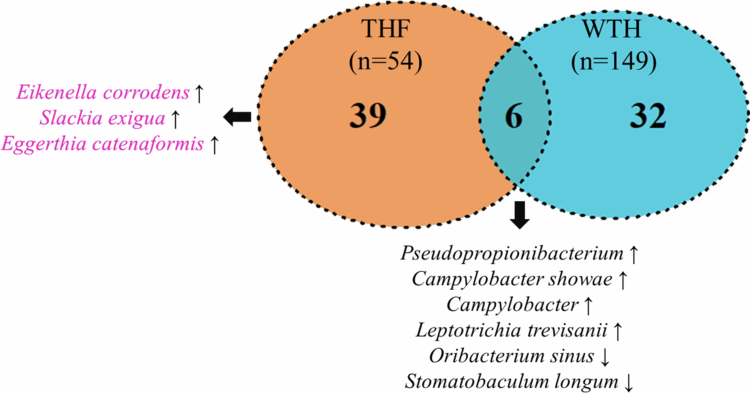
Differential composition profiles reveal relative bacterial abundances in tumour samples compared to normal samples. A positive fold change (log2 FC > 1) indicated an increased abundance of bacteria in tumour samples compared to matched normal samples, while a negative fold change (log2 FC < −1) signified a reduced abundance of bacteria in tumour samples. A Venn diagram summarises the number of bacteria with amplicon sequence variants differentially expressed exclusively in the THF group, exclusively in the WTH group, and shared by both groups.

Due to the lack of publicly available TGS datasets for WTH OSCC patients, we digitally extracted the V3–V4 portion from our TGS reads and compared the resulting profiles with three NGS-based OSCC datasets available in the public domain (PRJNA813634, PRJNA597251, and PRJNA362794). Differential abundance analysis across all four datasets (our THF set plus three WTH cohorts) identified 77 ASVs with |log₂ FC| > 1 between TPS and matched NPS samples. Of these, 39 ASVs were uniquely enriched in THF OSCC–most notably *E. corrodens*, *S. exigua*, and *E. catenaformis–*while 32 ASVs were exclusive to WTH OSCC. Six ASVs (including *Pseudopropionibacterium*, *Campylobacter showae*, *Leptotrichia trevisanii*, *O. sinus*, and *Stomatobaculum longum*) were shared between both groups (Figure 5). Detailed lists of significantly over- and under-represented taxa are provided in Tables S2 and S3, respectively. These findings demonstrate a clear divergence in bacterial composition between habit-free and habit-associated OSCC.

## Discussion

In this study, *E. corrodens*, *S. exigua*, and *E. catenaformis* emerged as the most diagnostically informative tumour-associated taxa. These species were identified through a two-step pipeline (sPLS-DA followed by RF feature selection), which consistently highlighted this three-species panel as optimal for distinguishing tumour from normal mucosa. Notably, although *S. exigua* and *E. catenaformis* were relatively low in abundance, they contributed disproportionately to model performance, underscoring the importance of predictive power beyond relative abundance. Importantly, cross-cohort comparisons revealed that all three species were uniquely enriched in the THF OSCC cohort and absent in WTH OSCC datasets, suggesting specificity to OSCC in patients without conventional carcinogenic exposures. Their diagnostic value remained robust even after accounting for gender differences, supporting their potential generalisability, though validation in larger cohorts is warranted.

Moving from biomarker identification to microbial community interactions, the co-occurrence network highlighted two divergent microbial clusters: a tumour-associated group (*E. corrodens*, *S. exigua*, *P. micra*, *G. morbillorum*) forming a dense, positively correlated community, consistent with polymicrobial synergy in promoting dysbiosis and inflammation, and a commensal group enriched in normal mucosa that was loosely connected and likely protective [33–35]. The segregation of these commensals from tumour taxa highlighting the ecological divergence between tumour-enriched and commensal taxa, which may underlie their distinct functional contributions.

To further link these ecological patterns with host biology, we explored microbial functional associations. Key tumour-associated species such as *E. corrodens*, *S. exigua*, *P. micra*, and *G. morbillorum* also served as hub taxa within the tumour module, showing strong positive correlations with other tumour-enriched microbes. Their centrality suggests coordinating roles in promoting dysbiosis and supporting tumour progression. Conversely, several species enriched in the adjacent clinically normal mucosa–*P. pallens*, *S. oralis*, and *O. sinus–*may play protective roles. *P. pallens* is a saccharolytic anaerobe proposed as a novel species and may contribute to carbohydrate fermentation that supports mucosal health [36,37]. *S. oralis* is an early oral coloniser with known mucin binding and epithelial adherence capabilities [38], while *O. sinus*, a member of *Lachnospiraceae*, was originally from sinus pus and is presumed to support anaerobic fermentation and local microbial diversity [39]. The enrichment in normal mucosa and segregation from tumour taxa in the co-occurrence network suggests contributions to a microbiome structure that resists dysbiosis and potentially delays carcinogenesis.

Our host–microbe functional correlation analysis further supports these ecological patterns. Tumour-associated taxa, including *E. corrodens* and *S. exigua*, were strongly linked to metabolic pathways such as lipid and glutamine–glutamate metabolism, both implicated in carcinogenesis through inflammation and metabolic reprogramming. Notably, the styrene degradation pathway was specifically linked to *E. corrodens*. Styrene metabolism has been reported to generate reactive intermediates capable of inducing oxidative stress and DNA damage, processes known to contribute to carcinogenesis [40]. This distinctive association suggests a potential, though indirect, mechanism through which *E. corrodens* may influence mucosal stress and tumour progression. Meanwhile, these taxa showed negative associations with normal-enriched pathways (e.g. butanoate and glutathione metabolism) that maintain epithelial integrity and immune homoeostasis [35,41–46]. These associations suggest that the identified microbes may contribute to, or reflect, metabolic remodelling within the tumour microenvironment.

Compared with earlier OSCC microbiome studies that relied on NGS of partial 16S regions, our use of full-length TGS enabled true species-level resolution. This technological advantage allowed us to uncover distinct taxa such as *E. corrodens*, *S. exigua*, and *E. catenaformis–*species that may have been masked at the genus level in short-read studies [14,16,18,29,47].

The tumour-enriched species identified in our study have been implicated in inflammation and microbial dysbiosis. *E. corrodens* is a common oral commensal frequently found in dental plaque and periodontitis, known to activate NF-κB signalling and pro-inflammatory cytokines, and has also been detected in OSCC [48,49]. *S. exigua*, though less abundant, is associated with periodontal disease [50], and promotes biofilm formation and chronic mucosal inflammation [51]. *E. catenaformis,* though less studied, has been isolated from the oral cavity and produces short-chain fatty acids such as butyrate which, in excess, may impair epithelial integrity and promote carcinogenesis [52–54]. Notably, its exclusive enrichment in THF OSCC suggests a tumour-permissive role. Consistently, KEGG analysis revealed enrichment of tumour-associated pathways, including pentose phosphate, HIF-1 signalling, one-carbon metabolism, central carbon metabolism in cancer, bacterial invasion, and base excision repair, highlighting potential mechanisms through which these species may contribute to carcinogenesis. Having established potential mechanistic roles, we next examined whether these tumour-enriched species were specific to our cohort or shared across populations.

To evaluate the specificity of the three-species panel, we compared V4-trimmed profiles across three independent WTH OSCC cohorts. Among 77 differentially abundant ASVs across datasets, 39 were exclusive to the THF cohort–including *E. corrodens*, *S. exigua*, and *E. catenaformis–*whereas only six were shared [28–30]. Previous studies have implicated *P. micra* in OSCC via periodontal inflammation [55–57], but the THF-specific enrichment of our three taxa suggests a distinct microbial aetiology in non-habit OSCC, supporting their value as population-specific biomarkers. Beyond cohort-level specificity, we also assessed whether these microbial signals carried clinical relevance across tumour stage and patient demographics.

Based on the clinical staging information summarised in Table 1, we evaluated whether the diagnostic species identified by TGS varied by tumour stage. Among the three most informative taxa, *E. corrodens* exhibited significantly higher abundance in late-stage tumours compared to early-stage cases (*p* = 0.023), suggesting a possible association with disease progression. In contrast, *S. exigua* and *E. catenaformis* did not show significant differences between early- and late-stage OSCC, indicating that their enrichment is likely established during early tumourigenesis (Table S1). This supports their potential utility as early diagnostic markers. Notably, the absence of stage-dependent variation for these species suggests that their microbial signals are already distinct from normal mucosa in stage I disease. This directly addresses the concern regarding whether early-stage tumour microbiomes are distinguishable from normal tissue using TGS. The consistent enrichment of *S. exigua* and *E. catenaformis* across all tumour stages underscores their diagnostic potential even in early, potentially more treatable stages of OSCC.

In addition to tumour stage, we also evaluated whether microbial biomarkers were associated with patient sex. *S. exigua* and *E. catenaformis* both exhibited gender-associated abundance patterns, with higher levels observed in male patients (*p* = 0.0097 and 0.0331, respectively; Table S1). However, their tumour-specific enrichment was consistently observed in both sexes, supporting their diagnostic relevance regardless of gender. *E. corrodens*, in contrast, showed no gender association (*p* = 0.95) but correlated with cancer stage, further emphasising its link to disease progression rather than demographic variation. These findings suggest that while host factors like gender may influence microbial abundance, they do not preclude the use of these taxa as general OSCC biomarkers. Nonetheless, incorporating host covariates such as gender in future predictive models may further enhance diagnostic precision. Together, these findings highlight their robustness as diagnostic markers, although certain caveats warrant consideration.

Nevertheless, we acknowledge several limitations of this study. The statistical power of our stage-stratified analyses is constrained by the modest cohort size, which may limit the ability to detect subtle microbial shifts across OSCC progression. The use of a single-centre Taiwanese cohort may also restrict generalisability. While full-length 16S rRNA sequencing enabled species-level taxonomic resolution, it does not capture strain-level variation or gene expression. Future integration of shotgun metagenomics and metabolomics will be necessary to clarify microbial functionality and host–microbe interactions.

Moreover, due to the strong site bias in our cohort–where the vast majority of tumours originated from the tongue–we were unable to evaluate microbial variation across different oral cavity subsites. Future studies with anatomically diverse OSCC cohorts are warranted to investigate site-specific microbial patterns and spatial heterogeneity within the oral tumour microbiome.

## Conclusion

Our high-resolution TGS of full-length 16S rRNA profiling in THF OSCC patients has revealed distinct species-level microbial signatures–most notably *E. corrodens,* S. exigua and *E. catenaformis–*that associate with pro-inflammatory and metabolic pathways and are absent in habit-associated OSCC cohorts. These findings advance our mechanistic understanding of microbiome-driven carcinogenesis in low-risk populations and establish a promising biomarker panel for early, non-invasive OSCC detection. Future longitudinal and multi-omics studies will be critical to translate these insights into clinical screening and prevention strategies. In summary, this study demonstrates that TGS-based microbiome profiling can reveal species-specific microbial shifts in THF OSCC, offering new avenues for non-invasive diagnosis and microbial-based prevention. Our findings support the development of predictive models and microbial biomarkers tailored to populations previously overlooked in traditional OSCC risk paradigms.

## Supplementary Material

Supplementary materials **Figure S1.** Rarefaction curves for microbial communities in OSCC. The rarefaction curve indicates the number of ASVs observed (y-axis) with different sequencing depths (x-axis) in this study.

Supplementary materials **Figure S2.** Composition of bacterial communities in NPS and TPS samples. (A) The relative abundance of top ten families. (B) The relative abundance of the top ten genera.

Supplementary materials **Table S1.** Correlation between microbial biomarkers and clinicopathological parameters.

Supplementary materials **Table S2.** Differential expressed bacteria in our dataset.

Supplementary materials **Table S3.** Differential expressed bacteria in three public datasets.

## Data Availability

All 16S rRNA gene sequence reads are available at the NCBI SRA under BioProject ID PRJNA1031593.
