# Supplementary material for: Novel biomarker identification for oral squamous cell carcinoma development in nonsmoker, nondrinker, and nonchewer patients using third-generation sequencing of oral microbiome
Source: J Oral Microbiol. 2025 Oct 2;17(1):2565452. doi: 10.1080/20002297.2025.2565452 (PMC12493606; doi:10.1080/20002297.2025.2565452)
Supplement: Supplementary materials — Figure S1. Rarefaction curves for microbial communities in OSCC. The rarefaction curve indicates the number of ASVs observed (y-axis) with different sequencing depths (x-axis) in this study. [file ZJOM_A_2565452_SM3085.docx]

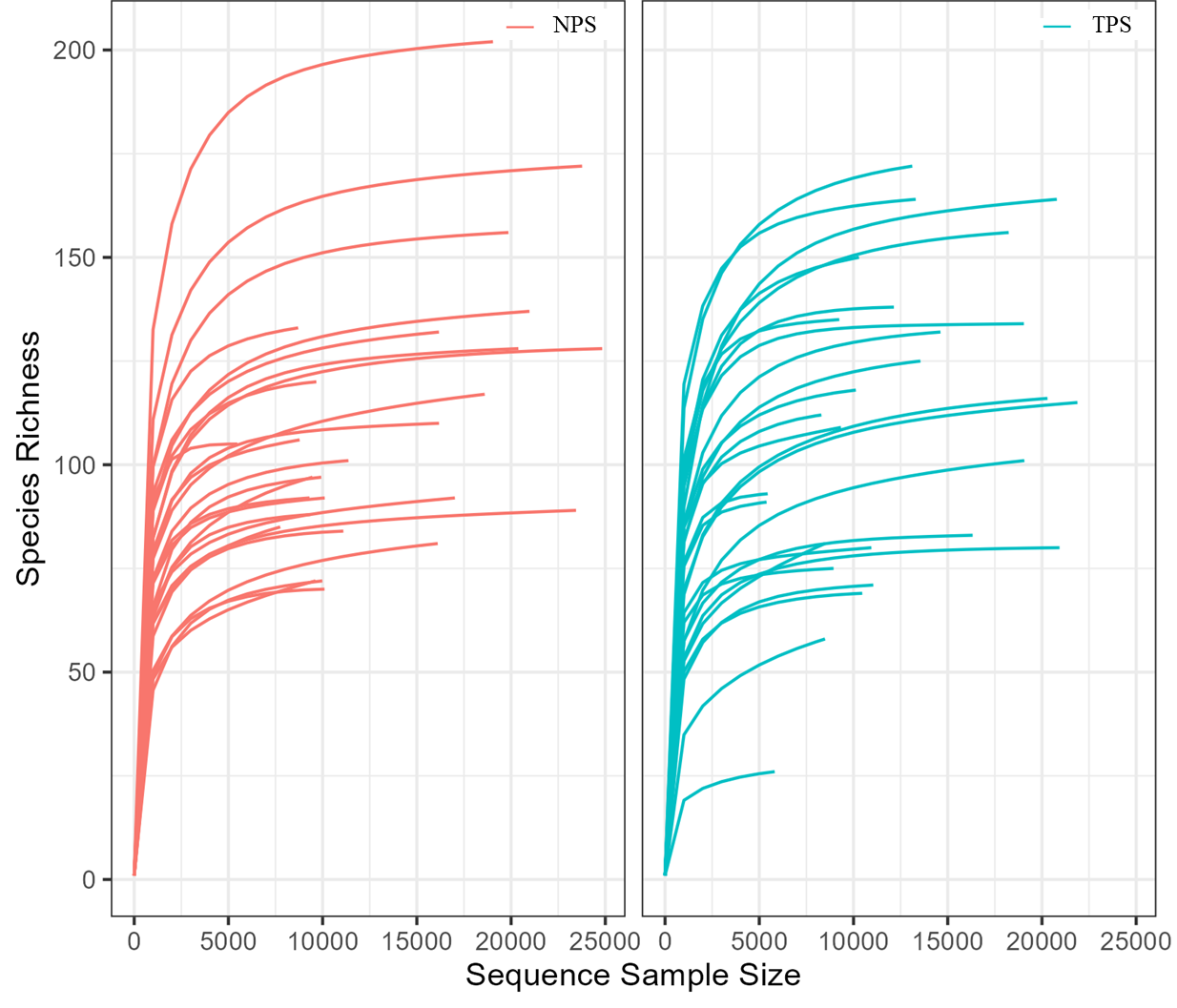


**Figure S1.** Rarefaction curves for microbial communities in OSCC. The rarefaction curve indicates the number of ASVs observed (y-axis) with different sequencing depths (x-axis) in this study.
