# Supplementary material for: Novel biomarker identification for oral squamous cell carcinoma development in nonsmoker, nondrinker, and nonchewer patients using third-generation sequencing of oral microbiome
Source: J Oral Microbiol. 2025 Oct 2;17(1):2565452. doi: 10.1080/20002297.2025.2565452 (PMC12493606; doi:10.1080/20002297.2025.2565452)
Supplement: Supplementary materials — Figure S2. Composition of bacterial communities in NPS and TPS samples. (A) The relative abundance of top ten families. (B) The relative abundance of the top ten genera. [file ZJOM_A_2565452_SM3086.docx]

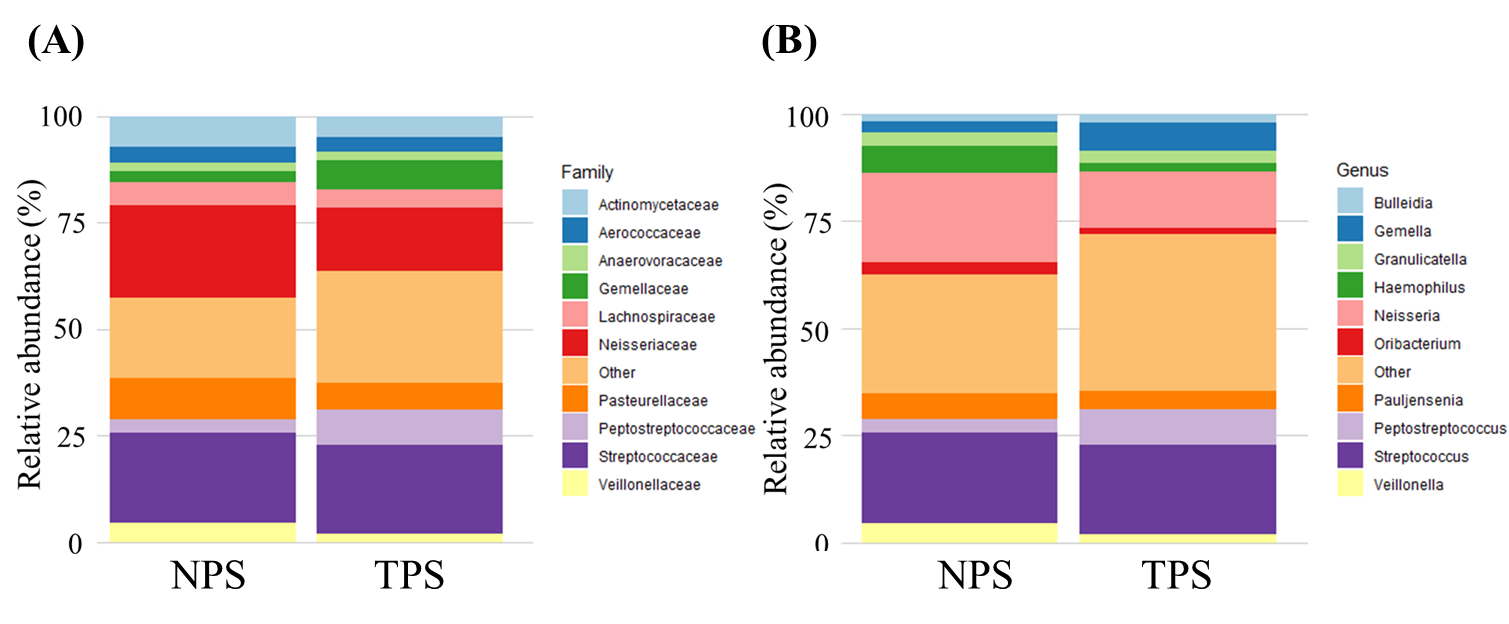


**Figure S2.** Composition of bacterial communities in NPS and TPS samples. (A) The relative abundance of top ten families. (B) The relative abundance of the top ten genera.
